# Prognostic value of lymphocyte-monocyte ratio at diagnosis in Hodgkin lymphoma: a meta-analysis

**DOI:** 10.1186/s12885-019-5552-1

**Published:** 2019-04-11

**Authors:** Shing Fung Lee, Ting Ying Ng, Devon Spika

**Affiliations:** 1 Department of Clinical Oncology, Tuen Mun Hospital, New Territory West Cluster, Hospital Authority, Tuen Mun, Hong Kong; 2 Health Economics Unit, Department of Clinical Science (Malmö), Lund University, Lund, Sweden; 3 Department of Economics, School of Economics and Management, Lund University, Lund, Sweden; 4 London School of Hygiene and Tropical Medicine, London, UK

**Keywords:** Meta-analysis, Lymphocyte, Monocyte, Hodgkin lymphoma, Prognosis

## Abstract

**Background:**

Prognoses of most adult Hodgkin lymphoma (HL) patients are excellent; most of them can achieve permanent remission that can be considered cured. However, many are under-treated or over-treated by standard modern therapies. An accurate determination of prognosis may allow clinicians to design personalised treatment according to individual risk of disease progression and survival. Lymphocyte monocyte ratio (LMR) at diagnosis has been investigated as a prognostic biomarker in patients with HL. Our objective with this meta-analysis was to explore the prognostic value of the LMR at diagnosis in adult HL, by investigating the association between LMR and survival outcomes.

**Methods:**

PUBMED and EMBASE were searched for relevant articles. Survival outcomes that we investigated included overall survival (OS), progression-free survival (PFS), event-free survival (EFS), lymphoma-specific survival (LSS), and time to progression (TTP). No restriction to the language, date, study country, or sample size was applied. Final search of databases was performed on 2 April 2018. We performed random-effects meta-analysis to aggregate and summarise the results from included studies, where four or more studies on a particular outcome were available.

**Results:**

A total of eight studies (all retrospective cohort studies) involving 3319 HL patients were selected for analysis. All studies except one reported the effect of LMR on OS; five reported on PFS, three reported on TTP and LSS, respectively, and one reported on EFS. The pooled estimates showed low LMR was associated with poor OS (hazard ratio [HR] 2.67, 95% CI 1.67, 4.26) and PFS (HR 2.19, 95% CI 1.46, 3.29). Subgroup analyses of OS stratified by LMR cut-off values and sample sizes both indicated that low baseline LMR was associated with poorer prognosis.

**Conclusions:**

Low LMR at diagnosis was associated with poor OS and PFS in HL. LMR is easy and cheap to determine and has a potential role in daily clinical management. More studies are needed to validate this biomarker and explore its interaction with known prognostic factors.

**Electronic supplementary material:**

The online version of this article (10.1186/s12885-019-5552-1) contains supplementary material, which is available to authorized users.

## Background

Hodgkin lymphoma (HL) is a type of lymphoma of B-cell origin. About 15% of lymphomas are HL [1]. Two major subtypes of HL are classical HL and nodular lymphocyte predominant Hodgkin’s lymphoma (NLPHL). Classical HL is further subclassified into four histological subtypes: nodular-sclerosis classical HL, lymphocyte-rich classical HL, mixed-cellularity classical HL, and lymphocyte-depletion classical HL [2]. HL is one of the most common malignancies in young adults aged 20–40 years [3]. Only about 15–35% of HL patients are older than age 60 years [3–6]. This variation in incidence by age at diagnosis is represented in Western countries by the bimodal age-incidence curve showing two peaks, first at around age 20 years, and second at around age 65 years [7]. While the bimodal curve is a defining epidemiological feature of HL, its shape varies significantly by race, socioeconomic status, geography, time, sex, and histological subtype [8–10]. For example, for calendar years 2010 to 2014 the age standardised incidence rates for Asian and Pacific islanders and non-Hispanic whites in the US were 1.2 and 2.9 per 100,000, respectively [9].

Standard treatment depends on stage and other clinical information. It usually consists of two to 8 cycles of chemotherapy followed by radiotherapy in selected patients [3]. The treatment algorithm is largely determined by clinical parameters such as age, stage and size of disease bulk. More than 95% of early stage HL, and up to 80–90% of intermediate or advanced stage patients can achieve permanent remission and can be considered cured with modern therapy [11]. However, at least 10–20% of patients in all stages may be under- or over-treated [11]. It is important at the time of diagnosis to determine the prognosis accurately. This allows clinicians to refine and tailor the treatment strategy, to avoid undertreatment (such as inadequate cycles of chemotherapy or exposure to ineffective cytotoxic agents) for patients at higher risk of disease relapse or increased resistance to chemotherapy and radiotherapy, and prevent overtreatment for those with a high chance of having their lymphoma cured, who may be suitable for less toxic therapies [11–13]. Patients who have their diseases relapsed after standard first line therapy need salvage treatment with high dose chemotherapy followed by autologous stem cell transplantation, and only half are successful treated [14]. On the other hand, HL long-term survivors have a two to four times increased risk of a second malignancy and cardiovascular disease compared with healthy members of the general population. This is important especially when most HL patients are young adults, and these long-term toxicities are associated with the anti-cancer treatment [15, 16]. One study evaluating the outcomes of decreased treatment intensity in a subgroup of early stage HL patients showed that the treatment effectiveness was not compromised when therapy was de-escalated, and more than 50% of all deaths during long-term follow-up were possibly related to the delivered treatment [17].

The goal of using prognostic markers to predict outcomes is to achieve a personalised approach: allowing us to provide more intensive or novel therapies (or avoid exposure to ineffective treatments) to patients with more aggressive disease, and to de-escalate therapy to patients with a high probability of achieving long-term remission, to spare the treatment toxicity.

The international prognostic score (IPS) is a standard stratification system for advanced classical HL [18]. Prognostic factors affecting clinical outcomes of NLPHL are similar to those included in the IPS [19–21]. For early stage HL, IPS is a less appropriate risk stratification system and other prognostic scoring systems can be used, such as those from the German Hodgkin Study Group (GHSG) [17], European Organisation for Research and Treatment of Cancer (EORTC) [22], and National Cancer Institute of Canada (NCIC) [23]. These prognostic systems are based mainly on clinical parameters, such as Ann Arbor staging, and tumour sizes [17, 18, 22, 23]. They do not consider the host immune status and tumour microenvironment, which can be variable among patients with similar clinical characteristics.

A gene expression profiling study has shown that a raised number of tumour-associated macrophages (TAMs) in a pre-treatment lesional tissue sample is associated with lower survival in patients with HL [24]. These macrophages are derived from peripheral blood monocytes [25] and have been positively associated with the percentage of peripheral blood monocytes [26]. They can secrete trophic factors (that affect tumour microenvironment), which have a role in the process where tumour cells interact with stromal and immune cells, including macrophages, B-cells and T-cells. The interaction in turn leads to neovascularisation and tumour growth [27–30]. Lymphocytes also have a role in immunosurveillance: the absolute lymphocyte count (ALC) has been shown to be a surrogate of host immune status and is an independent prognostic factor in HL [31].

Many of the advanced techniques for prognostication, including gene expression profiling [24] and immunohistochemical analysis [32, 33] have been studied. These are, however, costly and difficult to perform and interpret. A prognostic factor that is easily determined and widely available is needed.

Peripheral blood lymphocyte-to-monocyte ratio (LMR) at diagnosis may reflect the interaction between host immunity, represented by lymphocytes, and the tumour microenvironment, represented by monocytes. The peripheral blood count and cell count ratio can be determined readily and inexpensively by a standard automated complete blood count machine. Recent studies have indicated that peripheral blood LMR at diagnosis can predict long-term outcomes in haematological malignancies, including follicular lymphoma [34], diffuse large B-cell lymphoma [35], and NK/T cell lymphoma [36].

In HL, the consistency and magnitude of the prognostic value of LMR is controversial. Some studies have demonstrated a correlation between baseline LMR and survival outcomes, while one did not [37]. Different studies have, however, used different LMR cut-off values. One meta-analysis reported the prognostic value of LMR in various cancer types, and found that low LMR at diagnosis was associated with poorer cancer-specific survival and PFS in HL [38]. Since its publication in 2016, more studies on the prognostic role of LMR have been published, and three out of the seven analysed papers on HL were abstracts only. An updated analysis is needed to appraise and summarise the evidence.

This study aims to quantify the relationship between LMR at diagnosis and survival outcomes in adult HL, and to explore the impact of study characteristics on the prognostic value of LMR, by using meta-analytic techniques.

## Methods

In this study, we used meta-analysis to investigate the relationship between LMR at diagnosis and survival outcomes in adult HL. Specific outcomes considered were overall survival (OS), progression-free survival (PFS), event-free survival (EFS), lymphoma-specific survival (LSS), and time-to-progression (TTP). Definitions of the different survival endpoints are summarised in Table 1 [39].

**Table 1:** Definitions of survival endpoints

Endpoints	Definition
Overall survival	Entry into study until death as a result of any cause
Progression-free survival	Entry into study until lymphoma progression or death as a result of any cause
Event-free survival	Entry into study until any treatment failure including lymphoma progression, or discontinuation of treatment for any reason including death
Lymphoma-specific survival	Entry into study until time to death as a result of lymphoma
Time-to-progression	Entry into study until time to lymphoma progression or death as a result of lymphoma

Analysis and reporting were performed according to the Preferred Reporting Items for Systematic reviews and Meta-Analyses (PRISMA) and Meta-analyses of Observational Studies guidelines [40, 41]. Two reviewers (SFL and TYN) independently performed the literature search, assessed study eligibility, extracted the relevant data, and performed risk of bias assessment, following the strategy set out below. Any disagreement between the reviewers was resolved through discussion and consensus.

### Search strategy

Studies were identified through a systematic search of the EMBASE and MEDLINE databases, via the OVID platform. The search terms included “lymphocyte monocyte ratio” AND “Hodgkin lymphoma” (OR “Hodgkin’s lymphoma”). The search strategies for each database are described in the Additional file 1: Appendix S1. We did not apply restrictions to the language, date, study country, or sample size in our search. We performed the final search of all databases on 2 April 2018. Reference lists of relevant studies were also reviewed for possibly suitable articles. For non-English literature, we have used Google Translate for translation before we determined the eligibility for inclusion. Academic experts on lymphoma were contacted to identify any additional or unpublished data.

### Inclusion and exclusion criteria

Studies were considered eligible for inclusion if: 1) they reported data from an original, peer-reviewed study (i.e. not case reports, comments, conference abstracts, or review articles), 2) the study design included a prospective or retrospective cohort, case-control study, or randomised controlled trial, 3) they studied histologically proven HL in patients aged 18 years or older receiving primary treatment, 4) reported LMR as a dichotomised variable at diagnosis before specific anti-cancer treatment, 5) reported the prognostic outcome in terms of OS, PFS, EFS, LSS, or TTP, and 6) reported hazard ratios (HRs) of survival end-points according to high and low LMR with 95% confidence intervals (CI), or provided data for HR calculation. We excluded studies that did not provide quantification data or sufficient statistical parameters for analysis, or reported exclusively on patients aged below 18 years. We also excluded duplicate reports and studies covering overlapping populations. In cases where the same study population was reported on more than once, we included the most recent publication.

### Data extraction

We extracted information from the included studies on first author of the study, year of publication, journal, study design, country of study population, sample size, time period of study, median age and age range in the sample, sex distribution, cancer stage, IPS, median LMR, LMR cut-off value used, ratio of high to low LMR, survival outcomes investigated and hazard ratios (HR) for these, treatment modalities, and confounding factors adjusted for. We derived standard deviations and standard errors from the *p*-values, according to the instructions in the Cochrane Handbook for Systematic Reviews of Interventions [42].

### Quality and risk of bias assessment

We used the Quality In Prognosis Study (QUIPS) tool to assess the quality of each included study. The QUIPS tool was specifically developed for use in reviewing prognosis studies, which are prone to methodological challenges such as variation in methods and poor reporting, which may introduce important biases [43]. We assessed the quality of each study by evaluating the risk of bias in six domains: study participation and attrition, prognostic factor measurement, outcome measurement, confounding measurement, and statistical analysis and reporting. We assigned an overall grade for the risk of bias in the study (low, medium, high) based on the assessed risk of bias in each of the six domains. We adapted the QUIPS tool to the purpose of our analysis, by deciding a priori on the most relevant domains (outcome measurement, study confounding, and statistical analysis and reporting) to rate the overall risk of bias in the included studies [43]. We considered the inclusion of important potential confounding factors and the performance of multivariate analysis as important quality criteria for studies that investigated the prognostic significance of LMR in adult HL patients.

### Statistical analysis

This meta-analysis investigated the relationship between LMR at diagnosis and HL survival outcomes (OS, PFS, EFS, LSS, and TTP). The main summary statistics used in this meta-analysis were thus the relevant HRs for each survival outcome and their corresponding 95% CIs. Meta-analysis was conducted if a minimum of four studies were identified for a particular survival outcome. Heterogeneity between effect estimates was quantified. First, we determined the degree of between-study variability using the Cochran Q statistical test [44], we used a less conservative *p*-value of < 0.10 to indicate significant heterogeneity because of its low power when the number of studies is small [44]. Second, the I^2^ statistic was calculated to estimate the proportion of total variation across studies due to statistical heterogeneity but not chance [45]. I^2^ values of 25, 50, and 75% represent low, moderate, and high levels of heterogeneity, respectively.

We used a random-effects model to calculate a meta-analytic summary estimate of each HR, with 95% CIs, using DerSimonian and Laird’s method [46]. Random-effects meta-analysis takes into account statistical heterogeneity between studies, which can result from differences in the measurement of outcomes, interventions received by patients, or patient characteristics between studies [46]. Adjusted estimates were used in the analysis to account for confounding.

We performed subgroup analyses by LMR cut-off value and sample size, because it was expected that these factors would be different among studies and might potentially explain the varying survival outcomes. Sensitivity analyses were performed to examine statistical heterogeneity by omitting each study sequentially and assessing the effect estimate from remaining studies. Publication bias was assessed qualitatively using funnel plots of the logarithmic HRs versus their standard errors [47, 48]. We deemed the risk of publication bias to be low if the plot resembled a symmetrical inverted funnel [49]. We assessed publication bias quantitatively using the Egger regression test, and deemed publication bias as strongly suggested if *p* ≤ 0.10 [48].

All *p*-values were two-tailed. We considered a p-value of < 0.05 statistically significant, except when investigating heterogeneity and publication bias. All analyses and graphs were produced using Stata version 12 software (Stata, College Station, TX, USA) [50].

## Results

### Characteristics and quality of the included studies

**Fig. 1:**
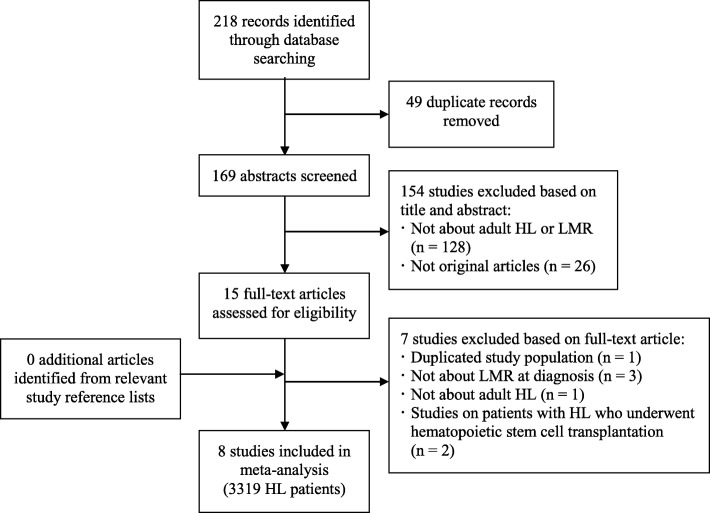
Flow diagram of study selection

Figure 1 presents a flowchart of the study inclusion process. We identified 218 studies from our literature search of the two databases. After removing 49 duplicates, we assessed 169 titles and abstracts, and excluded 154 records that did not meet the inclusion criteria. The full text of 15 citations was examined in detail. Nine studies fulfilled the inclusion criteria [26, 51–58], but two of them contained largely overlapping study populations [26, 53]. In this case, the more recent study with the larger population was selected for inclusion to avoid duplication [53]. In total eight studies involving 3319 HL patients were included in the meta-analysis [51–58]. Searching grey literatures, the reference lists of included studies and enquiring with academic experts on lymphoma did not identify further studies for inclusion. No unpublished relevant studies were identified. The conference abstracts and non-English literature that we did identify in our search were excluded because they did not contain enough information, had too short follow-up times, or were irrelevant.

Table 2 shows the characteristics of the included studies. All eight studies were published in English between 2012 and 2018 and all were retrospective cohort studies. All except one of these studies reported associations between LMR and OS [51–57]. Five reported on PFS [51, 52, 54, 55, 58], and three reported on TTP [51, 52, 56] and LSS [51–53], respectively. Only one study reported on EFS [57]. In all studies, HRs were estimated using Cox proportional hazards models for the associations between LMR and survival outcomes. A HR greater than one indicates a lower survival rate in patients with lower LMR. Seven studies were from the Western world and Israel [51, 52, 54–58], and one from Asia [53]. Median LMR among studies ranged from 2.1 to 3.3. In the study by Romano et al. [58], median LMR from healthy volunteers was described and it was 3.1 (range 0.6–4.0). LMR cut-off values and study sample sizes were not consistent across studies, LMR cut-off values ranged from 1.1 to 2.8 and sample sizes ranged from 101 to 1450. To determine the LMR cut-off values for analysis, studies used receiver operating characteristic curves and compared the sensitivity and specificity of the different cut-off values. Values having maximum joint sensitivity and specificity were selected. Other potential prognostic factors were investigated in some of the studies, including absolute monocyte count (AMC) [54], positron emission tomography (PET) [55], tumour associated macrophage (TAM) [57], and neutrophil-lymphocyte ratio (NLR) [58]. Some differences in the baseline characteristics such as stage and IPS in the LMR cut-off subgroups were noted in Porrata 2012b et al. [52], Koh et al. [53], Tadmor et al. [54], and Romano et al. [58]. However, these important confounders known to affect prognosis for HL have been adjusted for in the multivariable models.

**Table 2:**
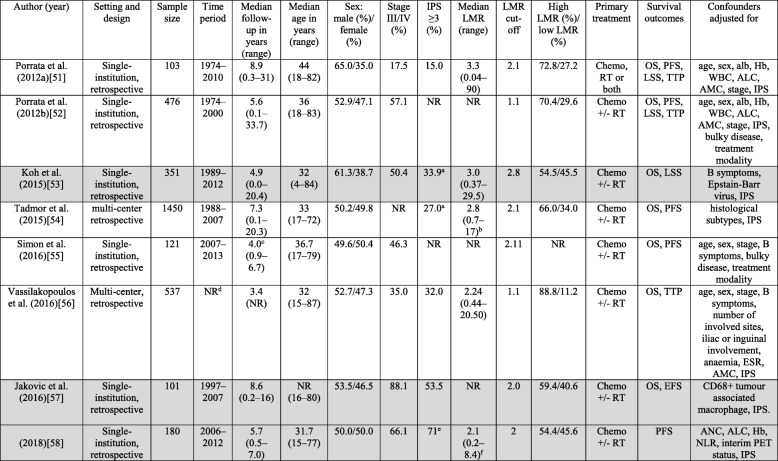
Characteristics of included studies assessing the LMR on prognosis in HL

Studies with a low overall risk of bias are shaded in grey

^a^Data were obtained by contacting the author

^b^2.5th to 97.5th percentile were stated instead of range

^c^Mean follow-up in years (range)

^d^The time period and range of follow-up in years were not reported

^e^IPS ≥2 (%)

^f^this study also provided median (range) of LMR of healthy control: 3.1 (0.6–4.0)

Abbreviations: *Alb* albumin, *ALC* absolute lymphocyte count, *AMC* absolute monocyte count, *ANC* absolute neutrophil count, *chemo* chemotherapy, *EFS* event-free survival, *Hb* haemoglobin, *HL* Hodgkin’s lymphoma, *HR* hazard ratios, *IPS* international prognostic score, *LMR* lymphocyte-monocyte ratio, *LSS* lymphoma-specific survival, *NLR* neutrophil-lymphocyte ratio, *NR* not reported, *OS* overall survival, *PET* positron emission tomography, *PFS* progression-free survival, *RT* radiotherapy, *TTP* time-to-progression, *WBC* white blood cell count

Overall, we assessed three of the included studies as having low risk of bias and five of the studies as having moderate risk of bias. Details of the risk of bias assessment are presented in Additional file 2: Table S1. The primary issues related to participant recruitment, because the exclusion and inclusion criteria were not always clear, and to statistical analysis and reporting, due to insufficient detail provided about methodology used (e.g. the rationale for selection of control variables and the model building strategy).

### Meta-analysis

Meta-analyses were conducted to investigate associations between LMR and OS and PFS, as these were the only survival outcomes for which four or more studies were identified.

#### Association between LMR and overall survival

**Fig. 2:**
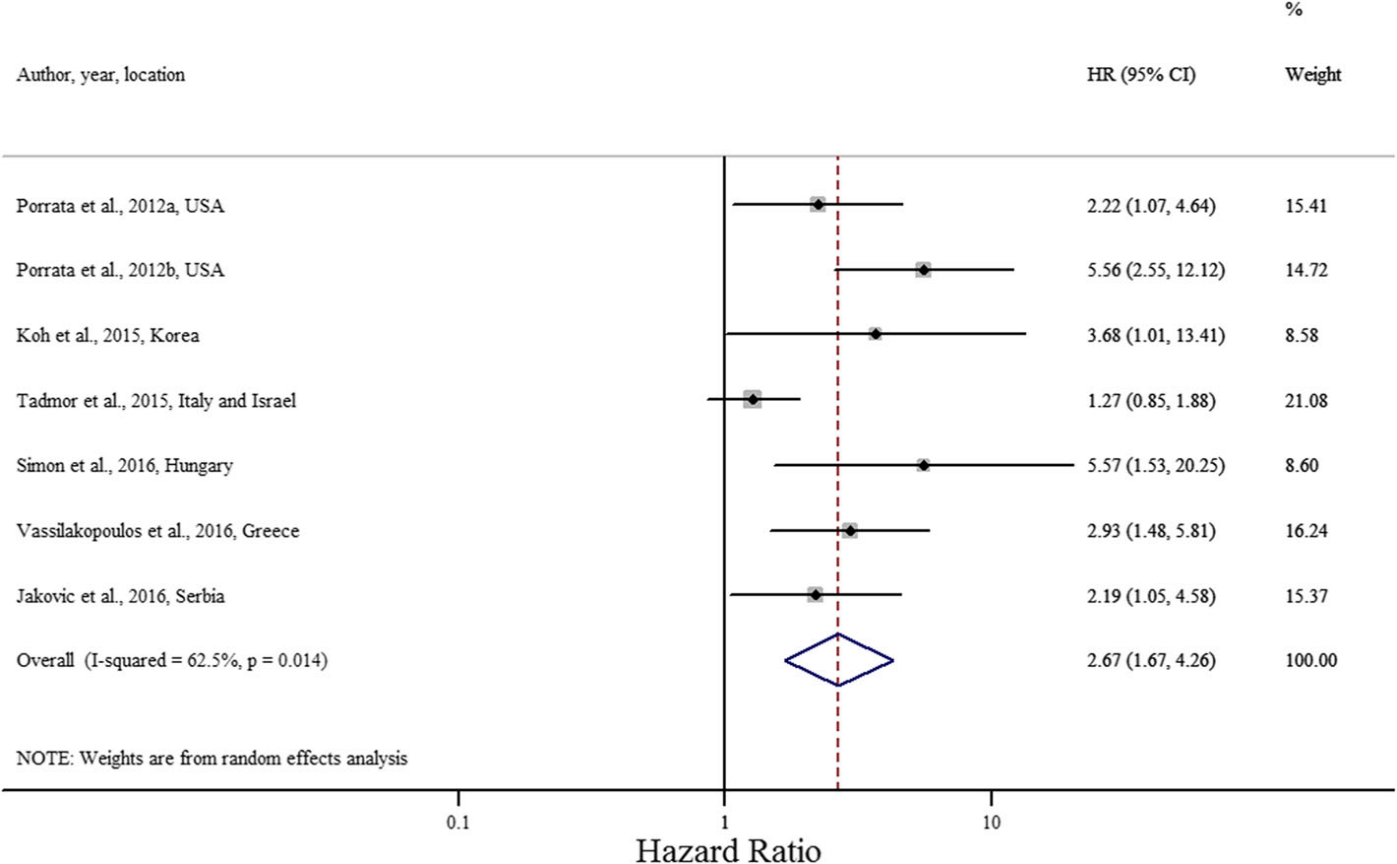
Forest plot evaluating HR of LMR on OS (*n* = 7)

In the meta-analysis, low LMR was associated with a significantly poorer OS, with a pooled HR of 2.66 (95% CI 1.67, 4.26; *P* = 0.014; I^2^ = 62.5%; Cochran Q test *P* = 0.014) (Fig. 2).

We conducted subgroup analyses by sample size and LMR cut-off value. Four studies had a sample size over 300 [52–54, 56], and four studies had an LMR cut-off value greater than two [51, 53–55].

**Fig. 3:**
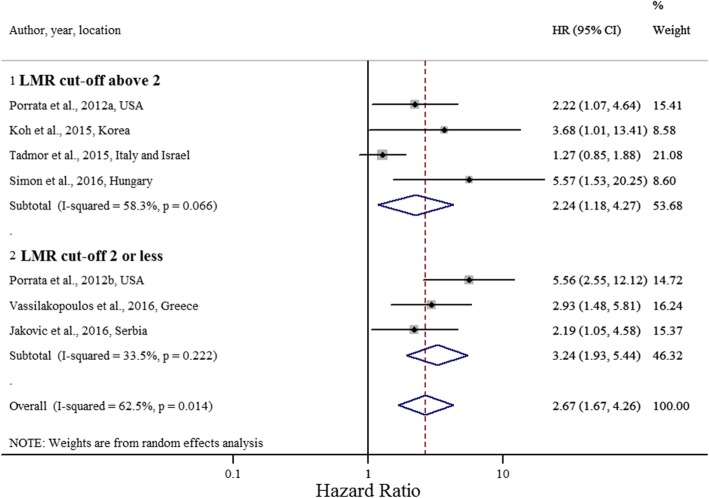
Forest plot evaluating HR of LMR on OS, stratified by LMR ratio cut-off value (*n* = 7)

**Fig. 4:**
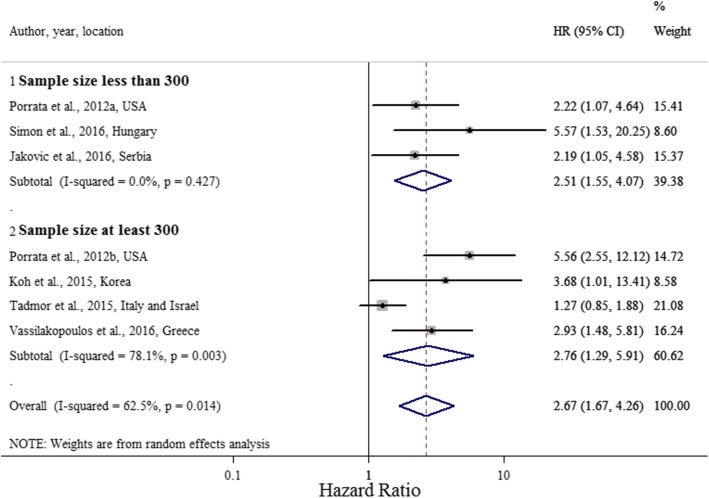
Forest plot evaluating HR of LMR on OS, stratified by study sample size (*n* = 7)

Studies with an LMR cut-off value greater than 2 had a pooled HR of 2.24 (95% CI 1.18, 4.27, *P* = 0.014; I^2^ = 58.2%; Cochran Q test *P* = 0.066) (Fig. 3), and studies with a sample size of 300 or more had a pooled HR of 2.76 (95% CI 1.29, 5.91, *P* = 0.009; I^2^ = 78.1%; Cochran Q test *P* = 0.003) (Fig. 4).

#### Association between LMR and progression-free survival

**Fig. 5:**
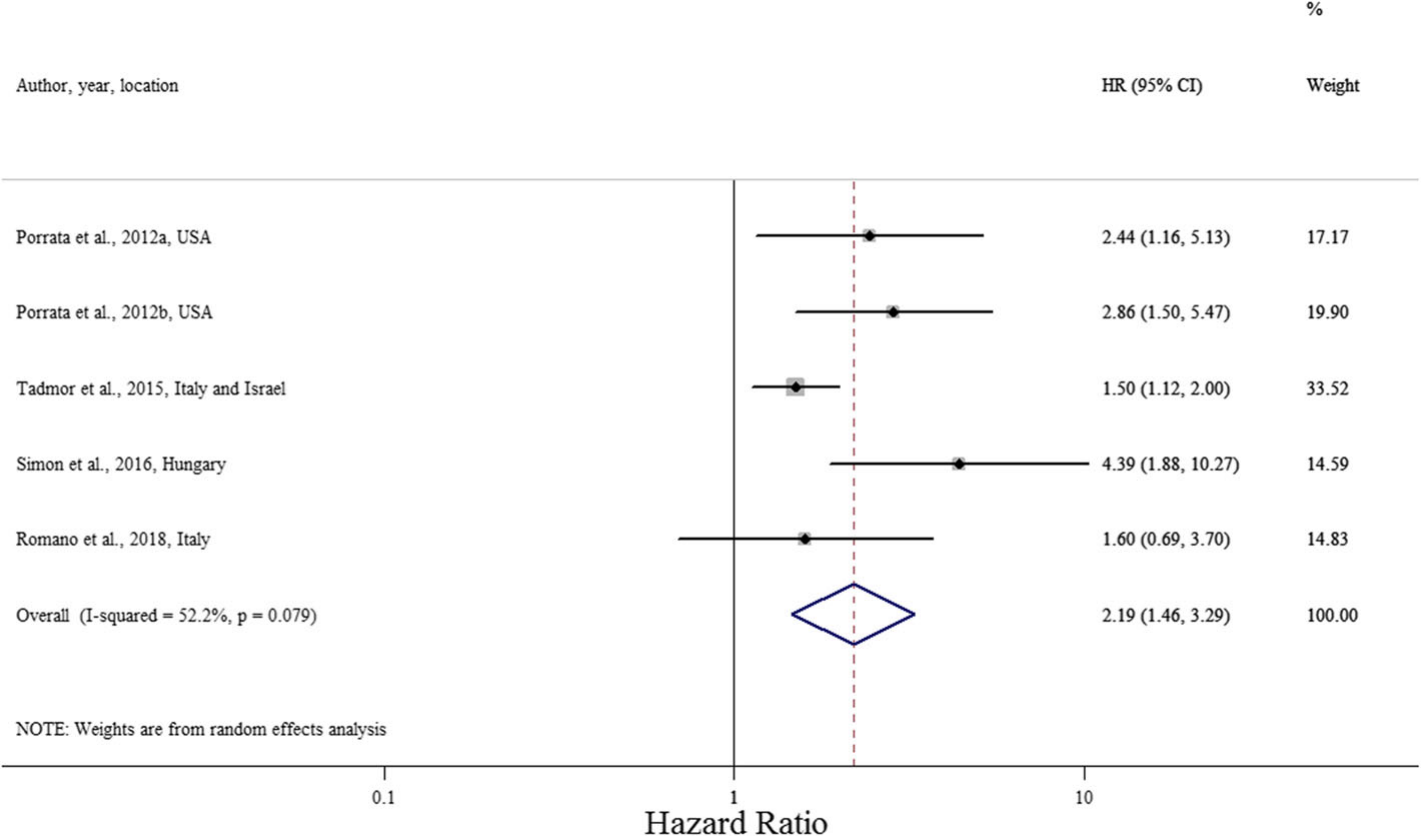
Forest plot evaluating HR of LMR on PFS (*n* = 5)

Five studies reported on the correlation between LMR and PFS (see Fig. 5) [51, 52, 54, 55, 58]. All were non-Asian studies [51, 52, 54, 55, 58], three had chosen an LMR cut-off value greater than 2 [51, 54, 55], and two had a sample size larger than 300 [52, 54]. The pooled estimate showed that low LMR was strongly associated with poorer PFS, with HR 2.19 (95% CI 1.46, 3.29, *P* < 0.001; I^2^ = 52.2%; Cochran Q test *P* = 0.079). Due to the lower number of analysable studies, subgroup analyses were not performed for PFS.

#### Sensitivity analyses and publication bias

We found that the results did not significantly change after omitting any of the included studies, demonstrating robustness of the results. The pooled HRs for OS ranged from 2.25 (95% CI 1.47, 3.43) to 3.12 (95% CI 2.22, 4.37). For PFS, the pooled HRs ranged from 1.86 (95% CI 1.34, 2.57) to 2.66 (95% CI 1.82, 3.88) (Table 3).

**Table 3:** Sensitivity analysis by sequential omission of each individual study

Meta-analysis estimates, given the named study is omitted		
Study omitted	Hazard ratio for overall survival (95% CI)	Hazard ratio for progression-free survival (95% CI)
Porrata et al. (2012a) [51]	2.82 (1.61, 4.93)	2.18 (1.33, 3.59)
Porrata et al. (2012b) [52]	2.25 (1.47, 3.43)	2.06 (1.29, 3.29)
Koh et al. (2015) [53]	2.60 (1.57, 4.31)	–
Tadmor et al. (2015) [54]	3.12 (2.22, 4.37)	2.66 (1.82, 3.88)
Simon et al. (2016) [55]	2.48 (1.53, 4.01)	1.86 (1.34, 2.57)
Vassilakopoulos et al. (2016) [56]	2.66 (1.53, 4.63)	–
Jakovic et al. (2016) [57]	2.82 (1.61, 4.94)	–
Romano et al. (2018) [58]	–	2.37 (1.45, 3.90)

**Fig. 6:**
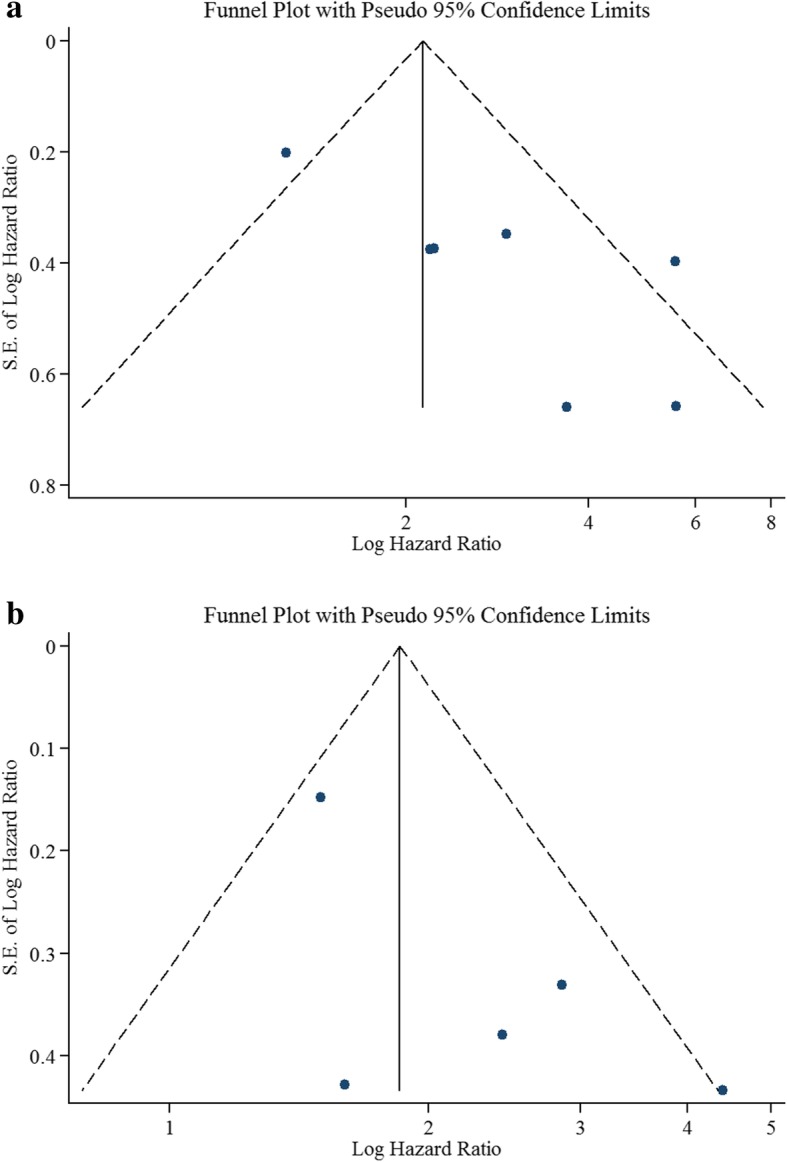
Funnel plot analysis of potential publication bias: **a** lymphocyte-monocyte ratio for overall survival; **b** lymphocyte-monocyte ratio for progression-free survival

We assessed publication bias visually using funnel plots (Fig. 6) and quantitatively using Egger’s test. We observe asymmetry in the funnel plot, suggesting publication bias for OS, as studies appear to be missing in the bottom left corner of the funnel plot. The null hypothesis for Egger’s test is that symmetry exists in the funnel plot. The *P*-values from the Egger test for OS and PFS are 0.022 and 0.117 respectively. There is therefore evidence to reject the null hypothesis at the 10% significance level for OS.

## Discussion

LMR may reflect the interplay between the host immunity and tumour microenvironment. The strong association identified through the meta-analyses between baseline LMR and survival outcomes indicates that LMR could be a good prognostic biomarker.

Low LMR at diagnosis may be related to more aggressive disease nature or to poorer tolerance to anti-cancer treatment. Low peripheral blood ALC and/or high AMC can cause low LMR. Lymphopenia, defined in IPS as < 0.6 × 10^9^/L or < 8% of total white cell, is a known prognostic factor for HL [18, 31].

This is compatible with our results because low ALC can lead to low LMR if the AMC is unchanged, since LMR is a ratio. Peripheral blood ALC is thought to be a surrogate of the host’s immunity status. On the other hand, the role of monocytes is different. One study showed that the peripheral blood AMC is positively associated with TAM content in lesional tissue samples, [26]. This is consistent with the evidence that TAMs originate from blood monocytes. TAM is important in cancer cell survival and proliferation and its quantity has been shown to be associated with poorer outcomes in a number of cancers [24]. Monocyte chemoattractant protein (MCP-1) belongs to the cytokine superfamily, and its binding with receptors triggers downstream signals that regulate macrophage adhesion, and recruit TAMs. MCP-1 has been associated with tumour growth and disease progression in several kinds of cancer [59]. Furthermore, a subpopulation of immune cells called monocytic myeloid derived suppressor cells (MDSCs) has been identified in marrow and peripheral blood. Their accumulation has been associated with cancer related inflammation and immunosuppression. MDSCs are also correlated with TAMs, and an increased amount of MDSCs confers a poorer prognosis in many cancer types including HL [60–62]. The available evidence thus demonstrates the prognostic role of non-malignant immune cells, and the possible utility of these in determining accurate prognoses.

This review has several strengths. The median follow-up periods in the included studies of patients with a well-defined disease entity were likely adequate (range 3.4–8.9 years), since most of the relapses of HL typically occur within the first 3 years [63]. LMR as a prognostic factor is biologically sound, and is determined by a simple, widely available blood test. In the subgroup analyses stratified by sample size and cut-off value, the prognostic value of LMR remained significant.

We do, however, need to be cautious in interpreting our results as all included studies were retrospective observational studies and it is possible that not all sources of heterogeneity have been adjusted for. Each included study had adjusted for some different covariables, and was conducted in different countries (one from Asia and seven from the Western world, including Israel). The differing ethnic groups and patient characteristics could be one reason for the variable effect sizes found. The optimal LMR cut-off values determined also varied across studies, possibly related to sample variability and differences in baseline patient characteristics. For example, a decrease in lymphocyte counts has been observed more frequently in the elderly [64]. The different treatment regimens (types and doses of chemotherapy agent) may also interact with other factors, such as age and performance status, resulting in variable survival outcomes.

An important limitation of this meta-analysis is that the number of included studies is modest, only eight. This limited the kinds of subgroup analyses we could perform because the number of available studies in some subgroups was low. For instance, subgroup analysis by study region could not be conducted because only one Asian study was available. This could have been useful because although ethnicity was not detailed in the studies, study regions could be surrogates of ethnic groups. It would have been useful to know whether ethnic (regional) differences modify the results, and if the findings can be applied to different ethnic groups.

Importantly, we do find evidence of publication bias in reporting of OS. Publication bias arises when the publication of findings is influenced by the nature and direction of the study results [65]. Although tests for publication bias are optimal only if the number of studies included in a meta-analysis is at least 10 [42], the interpretation of the effect estimate needs caution, as studies seem to be missing in the bottom left-hand side of the funnel plot (Fig. 6). Such studies would probably be trials with large standard errors and small sample sizes, with an overall survival for high LMR lower than that for low LMR. However, it is only an assumption that these studies were ever undertaken [66]. Asymmetry in a funnel plot can also result from poor methodological design in the identified studies, because poor methodological design typically results in the estimated effect size being spuriously magnified.

Potential problems with prognostic studies are manifold [67]. The most relevant ones for our study are: a) primary outcomes might not always be well defined, and b) there is no methodological and reporting standard, so that important information may be omitted and the importance of conclusions may be artificially inflated.

When assessing risk of bias and study quality we did find that five studies had a moderate risk of bias. Although each of the included studies had different risks of bias, their respective estimated HRs for OS and PFS were, however, similar at around 2–3. In the sensitivity analysis where we sequentially omitted each study, the results were largely stable, and no significant difference between studies of low risk and moderate risk was found.

Prior to conducting the pooled analysis, we assumed that effect sizes of the association between LMR and survival time may differ according to the methodological quality of the studies, so we decided to use a random-effects model for analysis. Furthermore, measures were taken to minimise the impact of other sources of reporting biases: we screened non-English papers and grey literature, including abstracts of studies presented at conferences and published in conference proceedings, and academic experts on lymphoma were consulted to identify further data. However, given the strong association between LMR and OS and PFS, respectively, and the observation that there was no significant change in the magnitude and direction of the pooled HRs in subgroup analyses and sensitivity analyses, the findings suggest that baseline LMR could be a significant prognostic marker in HL.

### Implications

Determination of protein (MCP-1) and immune cell subpopulations (TAM and MDSC) is costly, not available in routine daily clinical practice, and can be hard to interpret. On the contrary, measurement of LMR is easily performed. LMR is an inexpensive biomarker that can be determined by use of complete blood count machine, which is widely available worldwide. Data suggest that many aspects of standard care in modern medicine are lacking in developing countries and their treatment outcomes appear inferior in HL [68]. Initial imaging investigations may comprise chest X-rays and ultrasonography of the abdomen, which fall short of current standards [69]. Cell count ratios can be a simple complement that offers additional information in such situations.

Future directions for research include finding a unified cut-off value for HL, exploring the specific white cell subtypes (like B-lymphocyte and T-lymphocytes) and the value of their cell ratios in prognostic prediction, and investigating the combination of cell count ratios with established prognostic factors. The combination of different prognostic factors might be able to discriminate risks of disease progression and survival better. Finally, a prospective validation study should be conducted to confirm the clinical value of LMR and allow us to implement it in routine use.

## Conclusion

Low LMR at diagnosis is associated with poor OS and PFS. LMR therefore has the potential to be a practical biomarker that predicts survival outcomes in adult HL. A more accurate prediction in prognosis could allow clinicians to better tailor the treatment regimens according to individual risks. The test is inexpensive and easy to perform in outpatient or inpatient settings. Future prospective studies are needed to confirm its exact role in the disease management of HL, and the possibility of combining it with other established prognostic factors.

## Additional files

Additional file 1: **Appendix S1.** Electronic databases search strategy (DOCX 13 kb)

Additional file 2: **Table S1.** Risk of bias assessment of included studies using the Quality in Prognostic Studies tool. The six domains represent important issues to consider when evaluating the overall validity and bias in studies of prognostic factors. Some domains may not be relevant to the specific study. (DOCX 23 kb)

## Abbreviations

ALC: Absolute lymphocyte count
AMC: Absolute monocyte count
CI: Confidence interval
EFS: Event free survival
EORTC: European Organisation for Research and Treatment of Cancer
GHSG: German Hodgkin Study Group
HL: Hodgkin’s Lymphoma
HR: Hazard ratio
IPS: International prognostic score
LMR: Lymphocyte monocyte ratio
LSS: Lymphocyte specific survival
MCP-1: Monocyte chemoattractant protein-1
MDSCs: Monocytic myeloid derived suppressor cells
NCIC: National Cancer Institute of Canada
NLPHL: Nodular lymphocyte predominant Hodgkin’s Lymphoma
NLR: Neutrophil-lymphocyte ratio
NR: Not reported
OS: Overall survival
PET: Positron emission tomography
PFS: Progression-free survival
PRISMA: Preferred Reporting Items for Systematic Reviews and Meta-Analyses
QUIPS: Quality In Prognosis Studies
TAM: Tumour associated macrophage
TTP: Time to progression
