# Supplementary material for: Prognostic value of lymphocyte-monocyte ratio at diagnosis in Hodgkin lymphoma: a meta-analysis
Source: BMC Cancer. 2019 Apr 11;19:338. doi: 10.1186/s12885-019-5552-1 (PMC6458704; doi:10.1186/s12885-019-5552-1)
Supplement: Supplementary file 1 — Appendix S1. Electronic databases search strategy (DOCX 13 kb) [file 12885_2019_5552_MOESM1_ESM.docx]

**APPENDIX S1.** Search Strategy

**Search Strategy on Ovid MEDLINE**

1. ratio.mp. or blood cell ratio/

2. lymphocyte/

3. monocyte/

4. LMR.mp.

5. lymphocyte-monocyte ratio.mp.

6. lymphocyte to monocyte ratio.mp.

7. nodular lymphocyte predominant Hodgkin lymphoma/ or nodular sclerosis Hodgkin lymphoma/ or lymphocyte depletion Hodgkin lymphoma/ or lymphocyte-rich classical Hodgkin lymphoma/ or mixed cellularity Hodgkin lymphoma/ or classical Hodgkin lymphoma/ or lymphoma/ or lymphoma.mp.

8. 1 and 2 and 3

9. 4 or 5 or 6

10. 8 or 9

11. 7 and 10

**Search Strategy on EMBASE**

1. ratio.mp. or blood cell ratio/

2. lymphocyte/

3. monocyte/

4. LMR.mp.

5. lymphocyte-monocyte ratio.mp.

6. lymphocyte to monocyte ratio.mp.

7. nodular lymphocyte predominant Hodgkin lymphoma/ or nodular sclerosis Hodgkin lymphoma/ or lymphocyte depletion Hodgkin lymphoma/ or lymphocyte-rich classical Hodgkin lymphoma/ or mixed cellularity Hodgkin lymphoma/ or classical Hodgkin lymphoma/ or lymphoma/ or lymphoma.mp.

8. 1 and 2 and 3

9. 4 or 5 or 6

10. 8 or 9

11. 7 and 10
