# Supplementary material for: Prognostic value of lymphocyte-monocyte ratio at diagnosis in Hodgkin lymphoma: a meta-analysis
Source: BMC Cancer. 2019 Apr 11;19:338. doi: 10.1186/s12885-019-5552-1 (PMC6458704; doi:10.1186/s12885-019-5552-1)
Supplement: Supplementary file 2 — Table S1. Risk of bias assessment of included studies using the Quality in Prognostic Studies tool. The six domains represent important issues to consider when evaluating the overall validity and bias in studies of prognostic factors. Some domains may not be relevant to the specific study. (DOCX 23 kb) [file 12885_2019_5552_MOESM2_ESM.docx]

**Table S1.** Risk of bias assessment of included studies using the Quality in Prognostic Studies tool.

| **Author (year)** | **Study Participation^a^** | **Study Attrition^b^** | **Outcome measurement^c^** | **Confounding measurement^d^** | **Analysis and reporting^e^** | **Overall** |
| --- | --- | --- | --- | --- | --- | --- |
| Porrata et al. (2012a) [51] | Moderate | Low | Moderate | Moderate | Moderate | Moderate |
| Porrata et al. (2012b)[52] | Low | Low | Moderate | Moderate | Moderate | Moderate |
| Koh et al. (2015)[53] | Low | Low | Low | Low | Moderate | Low |
| Tadmor et al. (2015)[54] | Low | Moderate | Moderate | Low | Low | Moderate |
| Simon et al. (2016)[55] | High | Moderate | Low | Low | Moderate | Moderate |
| Vassilakopoulos et al. (2016)[56] | Moderate | Low | Moderate | Low | Moderate | Moderate |
| Jakovic et al. (2016)[57] | Low | Low | Low | Low | Moderate | Low |
| Romano et al. (2018)[58] | Low | Low | Low | Low | Low | Low |

Note: We rated “low risk of bias” for prognostic factor measurement domain in all studies, because the measurements were performed using standard complete blood count the same way to study participants of each study. No specific threshold for rating categories in QUIPS.

^a^ Source population adequately described, and study sample likely representative of usual Hodgkin lymphoma patients.

^b^ Infrequent loss-to-follow up, and the study data available (i.e., participants not lost to follow-up) adequately represent the study sample.

^c^ The outcome of interest is measured in a similar way for all participants. Study end-points well-defined.

^d^ Important potential confounding factors are appropriately accounted for.

^e^ The statistical analysis is appropriate and elaborated in detail. All primary outcomes are reported.
